# Supplementary material for: The gut microbiome in end-stage lung disease and lung transplantation
Source: mSystems. 2024 May 7;9(6):e01312-23. doi: 10.1128/msystems.01312-23 (PMC11237811; doi:10.1128/msystems.01312-23)
Supplement: Supplemental material — s and Methods; Figures S1 to S6. [file msystems.01312-23-s0001.docx]

**Supplementary Materials for**

**The Gut microbiome in End-stage Lung Disease and Lung Transplantation**

Shuyan Zhang, J. Casper Swarte, *et al.*

Corresponding author: Johannes R. Björk

**The file includes:**

Supplementary Materials and Methods

Figures S1 to S6

**Other Supplementary Material for this manuscript includes the following:**

Tables S1 to S10

**Supplementary Materials and methods**

**Study design**

**TransplantLines microbiome study**

We recruited patients with end-stage lung diseases and recipients who had undergone a lung transplantation. Their faecal samples were collected by the TransplantLines microbiome study (trial registration number NCT03272841). Eisenga et al. [1] has described the TransplantLines study in detail with regard to randomization, rationale of the study design and inclusion/exclusion criteria. We also included faecal samples collected from age-, gender- and BMI- matched renal donors from the TransplantLines cohort prior to the surgery as the healthy controls in this study.

**Ethical Approval**

An informed consent form was signed by all participants before sample collection. The institutional ethics review board in the University Medical Centre Groningen (UMCG) approved TransplantLines (METc 2014/077), which is in accordance with the UMCG Biobank Regulation and adheres to the World Medical Association Declaration of Helsinki and the Declaration of Istanbul.

**Clinical and laboratory characteristics**

At every study visit, a fixed set of laboratory parameters was measured and recorded into the study database with the consent of patients. Blood samples were collected in the morning after 8-12 hours of overnight fasting before the study visit and were measured by in-hospital routine assays to analyse clinical markers. Demographics and data on medication use were offered by the participants and confirmed by the patients during the study visit. Anthropometry measurements included height, body weight and fat percentage (multifrequency bioelectrical impedance device; BIA, Quadscan 4000, Bodystat, Douglas). General medical information at the time point of transplantation was extracted from electronic hospital records.

**Cross-sectional and longitudinal sampling**

The combination of collecting both cross-sectional and longitudinal samples made it possible to investigate the gut microbiome of patients with end-stage lung diseases, the short-term dynamics of the gut microbiome after lung transplantation and the long-term alteration of the gut microbiome after lung transplantation. Transplantation candidates are screened intensively before transplantation. Patients up to the transplantation standard were included in the TransplantLines study. Pre-transplantation patients were followed as end-stage disease patients. Further study visits were done at 3 months, 6 months, 12 months and 24 months after transplantation. Transplant recipients who underwent the transplantation before June 2015 were included in the cross-sectional part of the study for one study visit. The patients in the cross-sectional part of the study were not followed prospectively and were included at a time point > 1 year after transplantation. In this study, the mean delay of collecting cross-sectional samples is 6 years after lung transplantation; the numbers of longitudinal samples collected at 3 months, 6 months, 12 months and 24 months after transplantation are 5, 29, 34 and 17 respectively.

**Antibiotic use of lung transplant recipients**

Standard antibiotic regime peri-operatively is ceftazidim. However antibiotic selections are tailored to prior cultures obtained during full pretransplant assessment in all patients. For fungal profylaxis, we use nebulized amfotericine-B. Cotrimoxazol was used as PCP profylaxis. We do not administrate azitromycine as prophylaxis routinely direct post-operatively, but only when CLAD is diagnosed. After treatment with methylprednisolone for acute rejection, no additional prophylaxis is started. Patients continue cotrimoxazole, and it also depends on the time post-transplant valganclovir (3 months prophylaxis; D/R +/-, D/R +/+. 1 year prophylaxis D/R -/+). (Steroid) resistance acute rejection treatment with ATG valganciclovir prophylaxis is administrated for 4 months.

**Identification of medication regimens by unsupervised clustering**

According to standardised protocol, there are seven commonly used immunosuppressive drugs: tacrolimus, ciclosporin, mycophenolic acid, azathioprine, everolimus, sirolimus and prednisolone. Six antibiotic groups are frequently used: fluoroquinolone, penicillin, aminoglycoside, macrolide, imidazole and sulfonamide trimethoprim. Usage of these medications in LTR is listed in Table S1, and 1/0 was used to indicate whether the patient used/did not use the medication at the time point of the study visit. As described above, data about the use of immunosuppressive drugs and antibiotics is binary, so we first computed “Jaccard” dissimilarity indices using the vegdist function from the vegan R package. We then performed a hierarchical cluster analysis with the ward.D agglomeration method using the hclust function from the stats R package. We then plotted a dendrogram (Figure S1) showing the hierarchical relationships among samples from LTR. The height at which two samples are joined together represents the dissimilarity of their medication regimens. The larger the height, the more dissimilar their medication regimens. Samples joined together at height of zero were on the same medication regimen. We cut the dendrogram tree at the height of zero and five medication regimens with no less than five recipients were selected for further analysis.

**Generation of Microbiome data
Faecal sample collection and following processing**

FecesCatchers (TAG Hemi VOF) were sent to patients’ home for collecting the faecal samples the day before the TransplantLines study visit. Patients were asked to collect and store the faeces in appropriate tubes, which would be frozen at -18oC immediately after collection. The next day these frozen faecal samples were brought with patients in cold storage (with ice cubes or in a cooler) to the TransplantLines visit and then stored at -80oC until DNA extraction.

**DNA extraction**

QIAamp Fast DNA Stool Mini Kit (Qiagen) was used to extract microbial DNA following the instructions of the manufacturer with the help of the QIAcube (Qiagen) automated sample preparation system.

**Library construction and metagenomic sequencing**

For samples with total DNA amount lower than 200ng as measured by Qubit 4 Fluorometer, NEBNext® Ultra^TM^ DNA Library Prep Kit for Illumina was used for library preparation; while for samples with DNA yield lower than 200ng, NEBNext® Ultra^TM^ II DNA Library Prep Kit for Illumina® was used to prepare the libraries; according to the manufacturer’s instructions. Metagenomic shotgun sequencing was performed with the DNBSEQ Platform at MGI, China.

**Metagenomic data processing**

KneadData (v0.5.1) [2] was used to filter out adapters and low-quality reads (Phred score <30). Then reads aligned to the human genome (hg19) were removed using Bowtie2 (v2.3.4.1) [3]. Next the quality of the reads was assessed by FastQC toolkit (v0.11.7). In this study, the median numbers of reads before and after quality filtering are 54,517,354 and 51,041,118 respectively. Taxonomy alignment was performed by MetaPhlAn3 (v3.0.1) and Metacyc pathways were profiled by HUMAnN3 (v3.0.1) [4]. Antibiotic resistance genes were identified on short reads using shortBRED tool shortbred_quantify.py (v0.9.5), with markers generated using shortbred_identify.py (v0.9.5) on the comprehensive antibiotic resistance database (CARD) (<https://card.mcmaster.ca/>) of bacterial antibiotic resistance. As for the stringency criteria, we used the default parameter settings of the ShortBRED, i.e. 85% clustering identity and minimum marker length of 8 amino acids. Bacterial virulence factors were identified by shortBRED [shortbred_identify.py (v0.9.5) and shortbred_quantify.py tool (v0.9.5)] against virulence factors of pathogenic bacteria (VFDB) database (<http://www.mgc.ac.cn/VFs/main.htm>). Samples were further filtered out by criteria where eukaryotic or viral abundance > 25% of total microbiome content or total read depth < 10 million. In total, we identified 958 taxa (10 phyla, 22 class, 38 order, 71 family, 184 genera and 633 species), 536 metabolic pathways, 2255 virulence factors and 958 antibiotic resistance genes. With a prevalence threshold of 10%, 341 taxa (6 phyla, 15 class, 20 order, 35 family, 79 genera and 186 species), 303 metabolic pathways, 492 virulence factors and 357 antibiotic resistance genes were left after filtering. Analyses were performed using locally installed tools and databases on CentOS (release 6.9) on the high-performance computing infrastructure available at UMCG and University of Groningen (RUG). An example of scripts used for microbiome process is available at https://github.com/GRONINGEN-MICROBIOME-CENTRE/TransplantLines.

**Statistical analyses**

**Compositional data analysis**

High-throughput DNA sequencing produces compositional data in the form of relative abundances, which means that an observed increase of one microbial feature necessarily needs a decrease for another one [5]. Compositional data are strictly positive and vary from 0 to 1 if data are represented as proportions, or 0 to 100 if data are represented as percentages. However, standard statistical methodology is usually used for data that are represented by variables varying from -
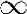
 to
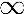
 within Euclidean space [6]. Therefore, a log-ratio transformation is needed to place the compositional data in a log-ratio coordinate space known as the Aitchison geometry in which standard statistical methodology can be used [5, 7]. After the transformation, the data about changes in the abundance of features can be obtained in the form of log-ratios with the denominator as the reference frame, which means that a change in the relative abundance of a given feature (the numerator) is always associated with a reference frame (the denominator) given by one or multiple other features [8].

**Aitchison PCA and PERMANOVA via adonis**

The Aitchison distance is the Euclidean distance between CLR-transformed compositions [6], and the CLR transformation is simply the centred log-ratio transformation, which can be obtained by


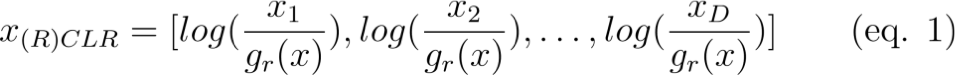


where x = [x_1_,x_2_,x_3_,...x_D_] denotes a sample consisting of D “counted” features (species or pathways), and g_r_(x) is the geometric mean defined on all observed features in the focal sample, and functions as the reference frame. The Aitchison distance meets all properties required for compositional data, including sub-compositional coherence and scale invariance [9]. Sub-compositional coherence ensures that the subset of analysed features does not influence the conclusions of the analysis; for instance, the results when only common features included will not change after the addition of rare ones. Scale invariance allows the analyst to treat two communities equally if they have the same relative abundances, even if they have different total abundances.

We performed permutational multivariate analysis of variance (PERMANOVA) tests on the Aitchison distances, to investigate whether different community configurations observed in the principal component analysis were statistically different from each other (for example, patients with end-stage diseases versus healthy controls).

**Differential abundance analysis on CLR abundances**

Differentially abundant microbial features were tested by modelling the CLR-transformed abundance of either microbial species or pathways using linear models with FDR correction. As described above, we modelled the CLR-transformed relative abundances of microbial species and pathways by linear models adjusting for confounders including age, sex and BMI. The following differential abundance analyses were performed: (1) patients with end-stage lung diseases (ESLD patients) before transplantation versus healthy controls; (2) lung transplant recipients after transplantation versus healthy controls; (3) lung transplant recipients after transplantation versus ESLD patients before transplantation; (4) ESLD patients and lung transplant recipients grouped by specific end-stage lung disease versus healthy controls; (5) compare the lung transplant recipients on different combinations of immunosuppressive drugs and antibiotics pairwise; (6) compare lung transplant recipients in different chronic lung allograft dysfunction stages pairwise; (7) compare the estimated average abundances of each microbial species between each consecutive time point; (8) compare the estimated average abundance of each microbial species in all time points (i.e. 3, 6, 12, and 24 months) after transplantation to its abundance before transplantation. In analysis (1)-(6), we used linear models (with the lm function in R) just including fixed effects, while in analysis (7) and (8), we used linear mixed models (with the lmer function from lmerTest R package) [10] including participant ID as a random effect. Specially for analysis (8), the estimated marginal means for the time point before transplantation were compared to the grand average of all time points after transplantation by the R package emmeans. Finally, we performed logistic regression on presence-absences of antibiotic resistance genes and virulence factors, both of which were extremely sparse in healthy controls. After each differential analysis, statistical significance was determined by correcting for multiple testing using the Benjamini-Hochberg false discovery rate (FDR) of 10% with the Padjust(..., n=n_features, method=”BH”) function in R.

**References for supplementary materials and methods**

1. Eisenga MF, Gomes-Neto AW, van Londen M, Ziengs AL, Douwes RM, Stam SP, Osté MCJ, Knobbe TJ, Hessels NR, Buunk AM, Annema C, Siebelink MJ, Racz E, Spikman JM, Bodewes FAJA, Pol RA, Berger SP, Drost G, Porte RJ, Leuvenink HGD, Damman K, Verschuuren EAM, de Meijer VE, Blokzijl H, Bakker SJL. Rationale and design of TransplantLines: a prospective cohort study and biobank of solid organ transplant recipients. *BMJ Open* 2018; 8: e024502.

2. McIver LJ, Abu-Ali G, Franzosa EA, Schwager R, Morgan XC, Waldron L, Segata N, Huttenhower C. bioBakery: a meta’omic analysis environment. *Bioinformatics* 2018; 34: 1235–1237.

3. Langmead B, Salzberg SL. Fast gapped-read alignment with Bowtie 2. *Nat Methods* Nature Publishing Group; 2012; 9: 357–359.

4. Beghini F, McIver LJ, Blanco-Míguez A, Dubois L, Asnicar F, Maharjan S, Mailyan A, Manghi P, Scholz M, Thomas AM, Valles-Colomer M, Weingart G, Zhang Y, Zolfo M, Huttenhower C, Franzosa EA, Segata N. Integrating taxonomic, functional, and strain-level profiling of diverse microbial communities with bioBakery 3. *eLife* 10: e65088.

5. Gloor GB, Macklaim JM, Pawlowsky-Glahn V, Egozcue JJ. Microbiome Datasets Are Compositional: And This Is Not Optional. *Frontiers in Microbiology* [Internet] 2017 [cited 2023 May 19]; 8Available from: https://www.frontiersin.org/articles/10.3389/fmicb.2017.02224.

6. Pawlowsky-Glahn V, Egozcue JJ. Compositional data and their analysis: an introduction. *Geological Society, London, Special Publications* The Geological Society of London; 2006; 264: 1–10.

7. Pawlowsky-Glahn V, Egozcue JJ. Geometric approach to statistical analysis on the simplex. *Stochastic Environmental Research and Risk Assessment* 2001; 15: 384–398.

8. Morton JT, Marotz C, Washburne A, Silverman J, Zaramela LS, Edlund A, Zengler K, Knight R. Establishing microbial composition measurement standards with reference frames. *Nat Commun* Nature Publishing Group; 2019; 10: 2719.

9. Aitchison J. On criteria for measures of compositional difference. *Math Geol* 1992; 24: 365–379.

10. Kuznetsova A, Brockhoff PB, Christensen RHB. lmerTest Package: Tests in Linear Mixed Effects Models. *Journal of Statistical Software* 2017; 82: 1–26.

**Supplementary Figures**

**
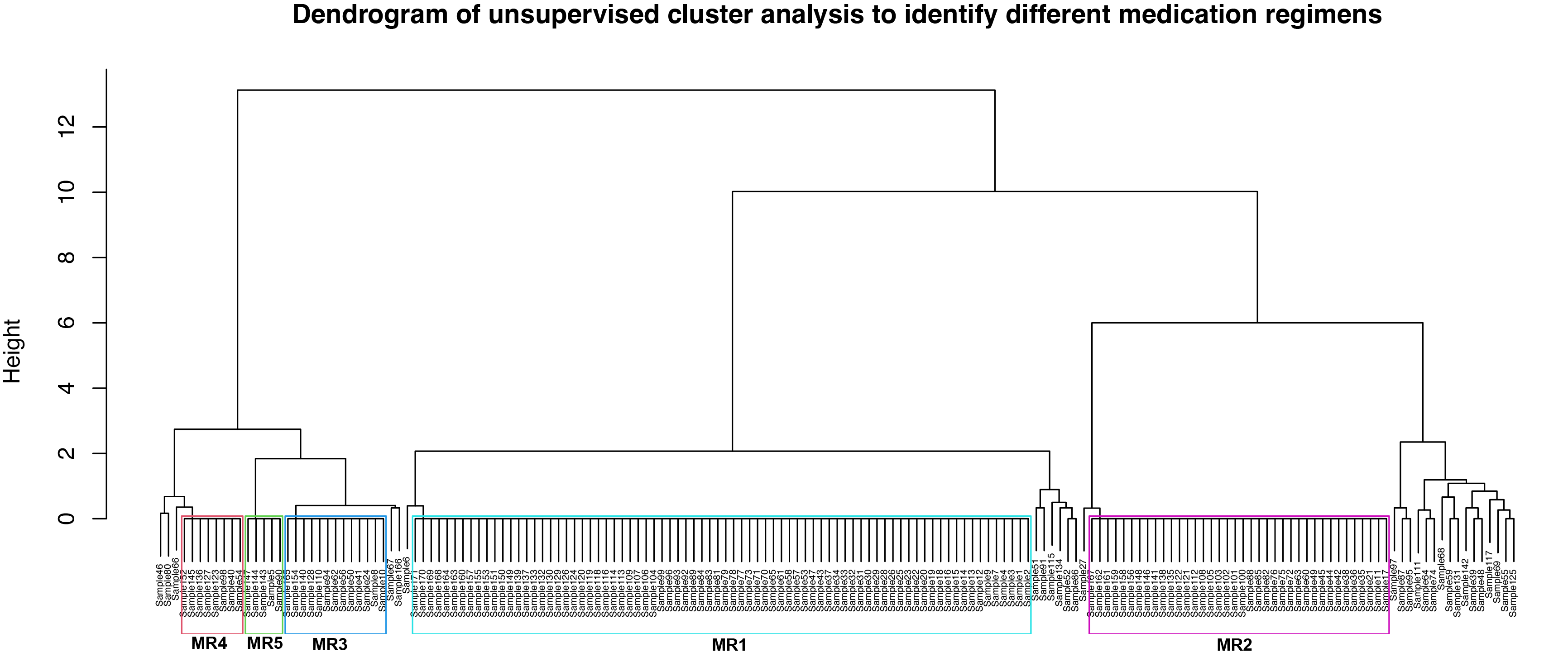
**

a)

b)

**Figure S1. Dendrogram of unsupervised cluster analysis to identify different medication regimens. (a)** Dendrogram shows the hierarchical relationship among samples from LTR. The height at which two samples are joined together represents the dissimilarity of their medication regimens. The larger the height, the more different the medication regimens. Samples joined together at a height of zero were on the same medication regimen. The dendrogram was cut at the height of zero, and medication regimens with no less than five recipients (different colour rectangles) were selected for further analysis. (b) By quantile-based grouping, LTR were categorized into five groups based on the time interval between sampling and lung transplantation, which was indicated by the colours of labels.


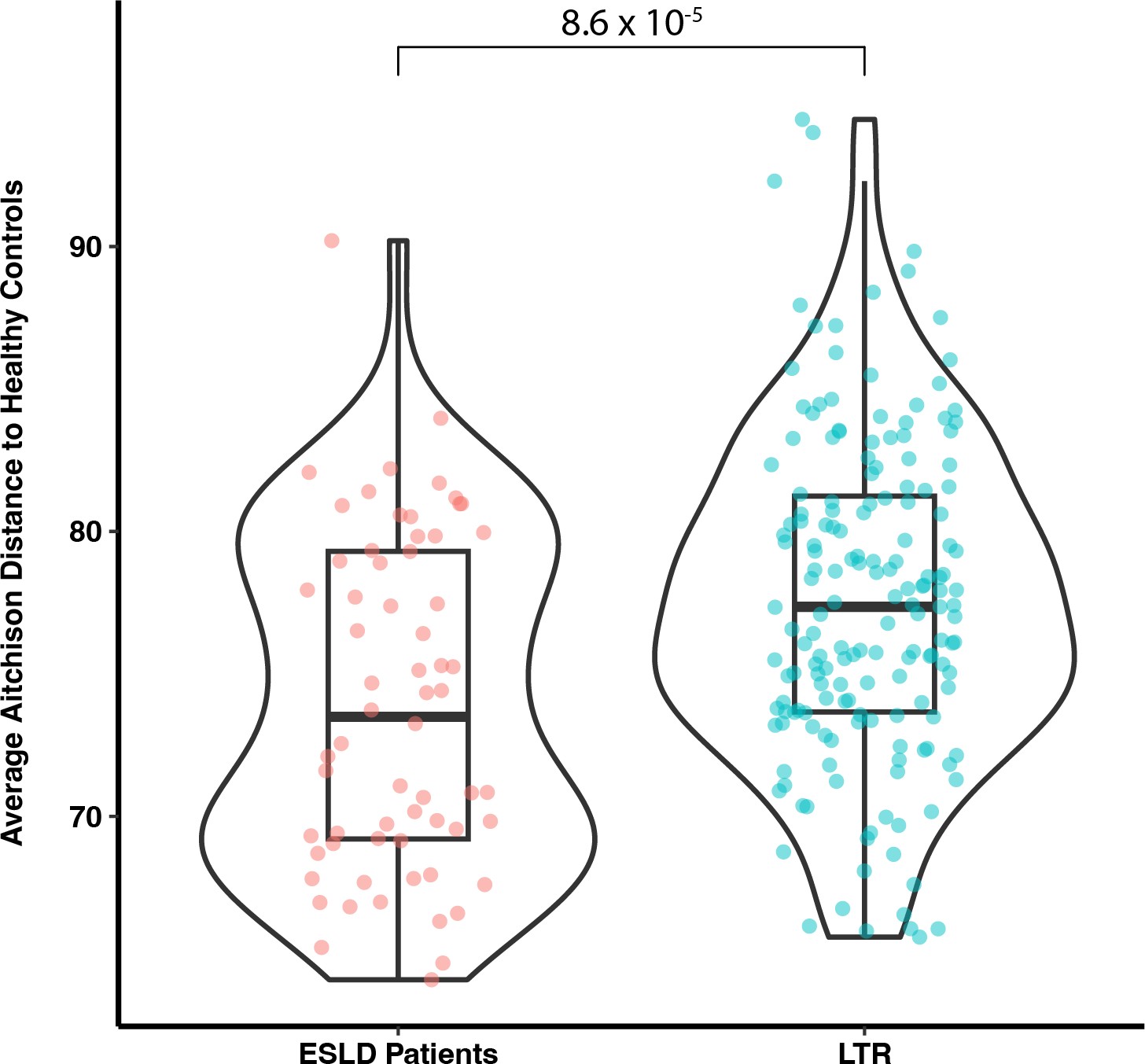


**Figure S2. The gut microbiome exhibits dysbiosis both before and after transplantation.** Violin plots shows the average Aitchison distance to the healthy controls of end-stage disease (ESLD) patients and lung transplant recipients (LTR). The value of each dot is the mean of the Aitchison distance of each sample to the healthy controls.

**Figure S3. Longitudinal diversity change of the gut microbiome after lung transplantation.** Smoothed conditional means plot displaying the longitudinal changes in Shannon diversity index of the gut microbiome in lung transplant recipients after transplantation (blue line). Shannon diversity index of the gut microbiome in HC was only measured at the timepoint of sample collection, shown as the green line for comparison.

**
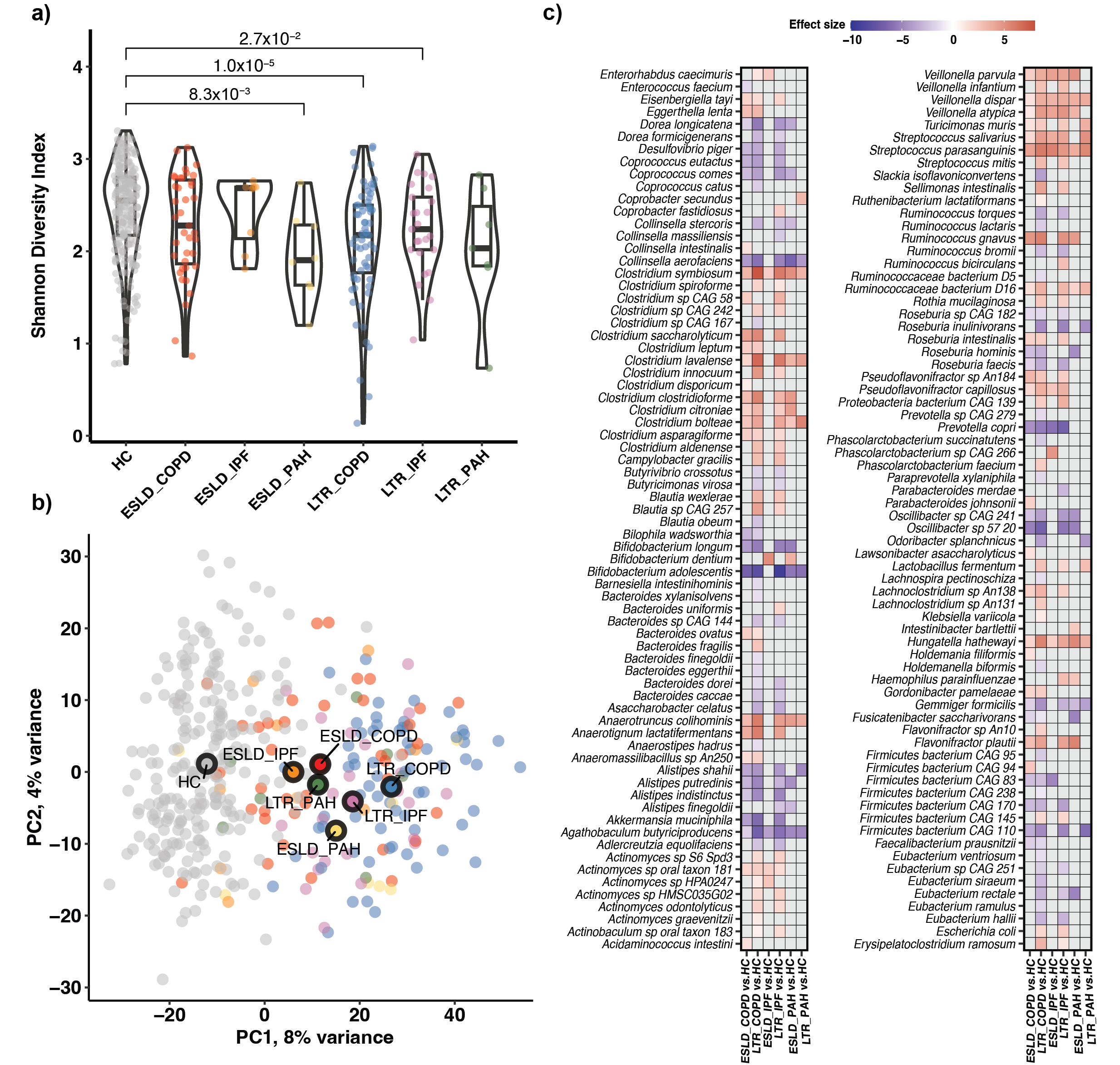
**

**Figure S4. Gut microbial signatures related to end-stage lung diseases.** (**a**) Violin plots depict the Shannon diversity index of patients with different end-stage diseases (indicated by ESLD followed by the specific disease), lung transplant recipients treated for different end-stage lung diseases (indicated by LTR followed by the specific disease) and healthy controls (HC). (**b**) PCA scatter plot with samples as dots: healthy controls in grey, COPD patients in red, IPF patients in orange and PAH patients in green. Blue, purple and yellow dots represent samples from LTR treated for COPD, IPF and PAH, respectively. The seven larger circles represent the centroid of each group. The distance is the Aitchison distance, and samples closer to each other have more similar gut microbial community compositions. (**c**) Heatmap shows the Log-fold changes (i.e. effect sizes) of differentially abundant microbial species in patients with COPD or LTR treated for COPD, IPF and PAH compared with HC. Cells in red/purple indicate positive/negative Log-fold changes (i.e. effect sizes), which means the focal species showed higher/lower relative abundance in patients with specific end-stage disease or LTR treated for specific end-stage disease compared with HC. Cells in grey indicate that the focal species showed no significant difference in the corresponding comparison.


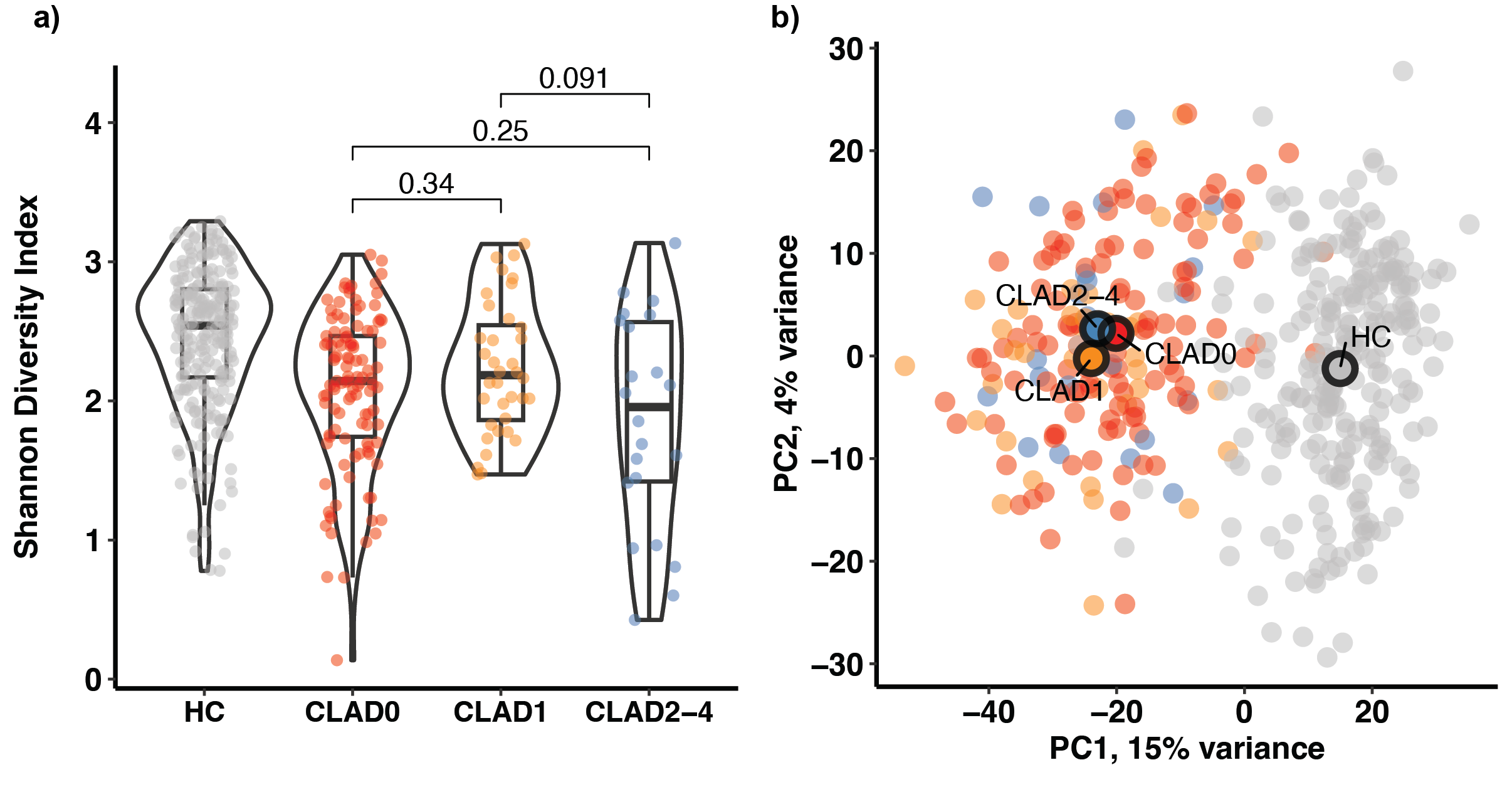


**Figure S5. Shannon diversity index and two principal components of gut microbiome in LTR for different CLAD stages and healthy controls.** (**a**) There was no significant difference in Shannon diversity among LTR with different CLAD stages (Mann-Whitney U, PCLAD0 vs. CLAD1=0.34, U=1820; PCLAD0 vs. CLAD2-4=0.25, U=1436; PCLAD1 vs. CLAD2-4=0.091, U=502). (**b**) The gut microbial composition of recipients with CLAD0 stage was significantly different from those at with CLAD1 stage (PERMANOVA: PCLAD0 vs. CLAD1=0.030), while the differences of gut microbial β-diversity in other two comparisons were not significant (PERMANOVA: PCLAD0 vs. CLAD2-4=0.339; PCLAD1 vs. CLAD2-4=0.127).


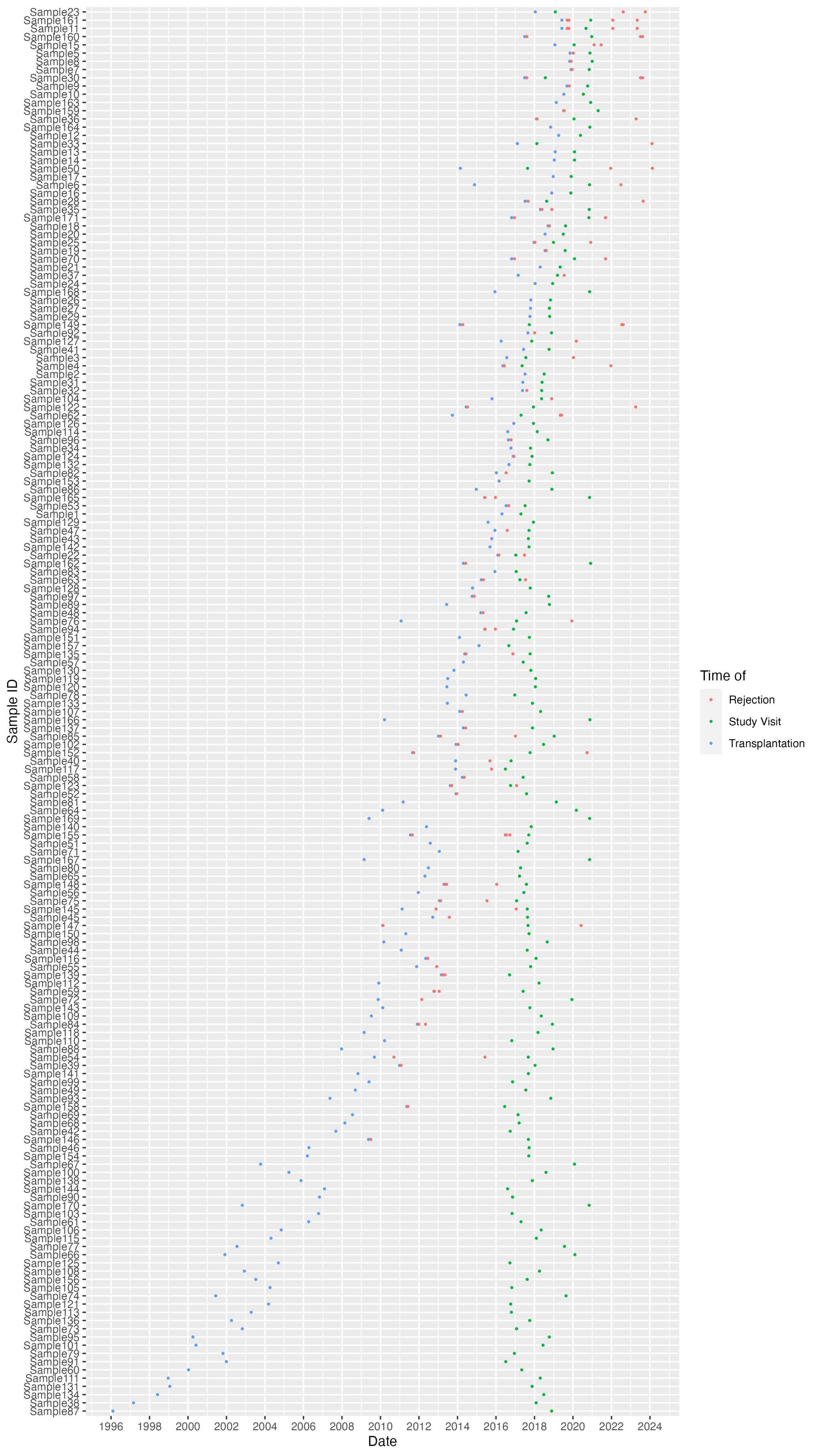


**Figure S6. Dates of lung transplantation, study visit and rejection occurrence for each lung transplant recipients (LTR).** Dots in the same row of the scatter plot indicate the dates of three events for the same LTR: Rejection in red, study visit in green and transplantation in blue.
